# The capacity of inspection on farm and at the abattoir to predict post‐mortem outcomes in slaughter pigs: A study at animal level

**DOI:** 10.1111/asj.13798

**Published:** 2022-12-30

**Authors:** Dayane Lemos Teixeira, Laura C. Salazar, Rafael Larraín, Laura A. Boyle

**Affiliations:** ^1^ Department of Animal and Agriculture Hartpury University and Hartpury College Gloucester UK; ^2^ Instituto de Ciencias Agroalimentarias, Animales y Ambientales (ICA3) Universidad de O'Higgins San Fernando Chile; ^3^ Departamento de Ciencias Animales Pontificia Universidad Católica de Chile Santiago Chile; ^4^ Pig Development Department Teagasc Animal and Grassland Research and Innovation Centre, Moorepark Fermoy, Co. Cork Ireland

**Keywords:** ante‐mortem, meat inspection, pigs, post‐mortem, welfare

## Abstract

The aim of this study was to investigate associations between ear, tail, and skin lesions, hernias, bursitis, and rectal prolapses at the abattoir and meat inspection outcomes in slaughter pigs, including carcass condemnations and trimmings, carcass weight, and carcass quality. This was an observational study whereby pigs were managed according to routine practices in a single abattoir. Data were collected from 1816 pigs. The relationship between animal‐based welfare and post‐mortem outcomes was analyzed using generalized mixed models (Proc Glimmix). Our findings showed that tail lesions were associated with entire carcass condemnations and trimmings (*P* < 0.001), a reduction in carcass weight (*P* < 0.05), and a potential to impair carcass quality by reducing muscle pH (*P* < 0.05), especially in carcasses from male pigs (*P* < 0.05). Additionally, hernias were associated with viscera condemnation (*P* < 0.05) and a reduction in carcass weight (*P*  < 0.05). Therefore, our findings confirm that ante‐mortem inspection could be useful to predict post‐mortem outcomes in the same pigs, especially in cases of tail lesions and hernia, which might trigger attention of the veterinary inspector in charge of the post‐mortem inspection.

## 1 INTRODUCTION

This research was part of a broader study evaluating animal‐based welfare outcomes in slaughter pigs assessed on farm and at the abattoir to support the use of ante‐mortem and post‐mortem meat inspections as an animal health and welfare diagnostic tool. The study was focused on evaluating the prevalence of ear, tail, and skin lesions, hernias, bursitis, and rectal prolapses in pigs, as well as associated on‐farm risk factors, association with post‐mortem inspection outcomes and financial implications. These animal‐based welfare outcomes were chosen due to their relevance to the pig sector but also because they are visible animal‐based welfare outcomes easily detectable on farm and at ante‐mortem and post‐mortem inspections.

To discover the association between the presence of these animal‐based welfare outcomes at ante‐mortem inspection (both on farm and at the abattoir) and post‐mortem inspection outcomes is useful to address the organization of official controls in abattoirs (Ghidini et al., 2021). Pigs affected by gross abnormalities have an increased probability of showing post‐mortem abnormalities (Harbers et al., 1992) and have a higher probability of having meat rejected at post‐mortem inspection (Jackowiak et al., 2006). Therefore, on‐farm assessments can complement (Teixeira et al., 2020) or facilitate (rather than replace) abattoir‐based ante‐mortem inspection by segregating sick or injured animals and animals with impaired welfare prior to transport into groups with and without visible lesions (Harbers et al., 1992). It can also prevent animals that are not “fit” from being sent to the abattoir for slaughter (EFSA, 2011) thereby protecting animal welfare.

Unhealthy pigs that have, or are suspected of having, injuries or illnesses on farm may be housed in “hospital,” “recovery,” or “isolation” pens to help improve their condition and/or to avoid infecting healthy animals, with on‐farm euthanasia as an alternative for sick/infected pigs in extreme situations. Knowing the likelihood that the carcass of these sick or injured animals might be condemned could also justify earlier euthanasia on farm, which would help avoid financial losses associated with treatment and reduced feed efficiency, misuse of antibiotics, and unnecessary animal suffering.

In the case of ante‐mortem inspection during unloading or in lairage at the abattoir, animals can be detained for closer examination if there are concerns regarding animal health and welfare. Usually, it must occur within 24 h of animals arriving at the abattoir, and slaughter must occur within 24 h of the ante‐mortem inspection. Ante‐mortem inspection is important to enable early detection of clinically observable zoonotic diseases and animal identification enabling traceability and evaluation of visual cleanliness of animals (EFSA, 2011). It is also essential for detecting conditions that cannot be detected at post‐mortem inspection and detection of animal welfare conditions (Stark, 1996), such as lameness (EFSA, 2011). Therefore, the veterinary inspector (VI) in charge of ante‐mortem inspections at the abattoir can advise the VI on the slaughter line that some pigs or batches require more thorough inspection rather than just a visual inspection.

The ability of data obtained from the ante‐mortem inspection to predict post‐mortem outcomes was investigated by Ghidini et al. (2021). However, Ghidini et al. (2021) assessed conditions typically found during ante‐mortem and post‐mortem inspections in slaughter pigs at batch level, and unfortunately, their pigs were not followed individually during ante‐mortem and post‐mortem inspections. Research is lacking on the association between the animal‐based welfare outcomes that can be detected on farm, at ante‐mortem and post‐mortem inspections, and the associated post‐mortem outcome at individual level. Therefore, the aim of this study was to investigate associations between ear, tail, and skin lesions, hernias, bursitis, and rectal prolapses at the abattoir and meat inspection outcomes in slaughter pigs, including carcass condemnations and trimmings, carcass weight, and carcass quality.

## 2 MATERIALS AND METHODS

This study was part of a research project approved by the Scientific Ethics Committee for Animals and Environmental Care of the Pontificia Universidad Católica de Chile (protocol number 170529006) and by the Research Department Ethic Committee of the Universidad de O'Higgins (No. 002‐2020).

This was an observational study whereby pigs were managed according to routine practices in a pig abattoir located in Chile. Data were collected from November 2018 to April 2019. On each day, data were collected from 9:00 h to approximately 15:00 h. Two observers (a veterinarian and a veterinary technician) were trained before the beginning of the study to ensure interobserver reliability, and the study consisted in two phases described below.

### 2.1 Phase 1: Animal‐based welfare outcomes, viscera condemnations, and cold carcass weight

The first phase of the study was carried out during 24 slaughter days using a convenience sample of 1500 slaughter pigs. The methodology was based on Teixeira et al. (2016), and data were collected at three points on the slaughter line: (I) before dehairing and evisceration; (II) at post‐mortem meat inspection; and (III) at the weighing scales. To avoiding biosecurity issues associated with moving between collection points on the slaughter line, cleaning and sanitation programs were in place to ensure all the hygiene standard required in a plant manufacturing meat products.

**FIGURE 1:**
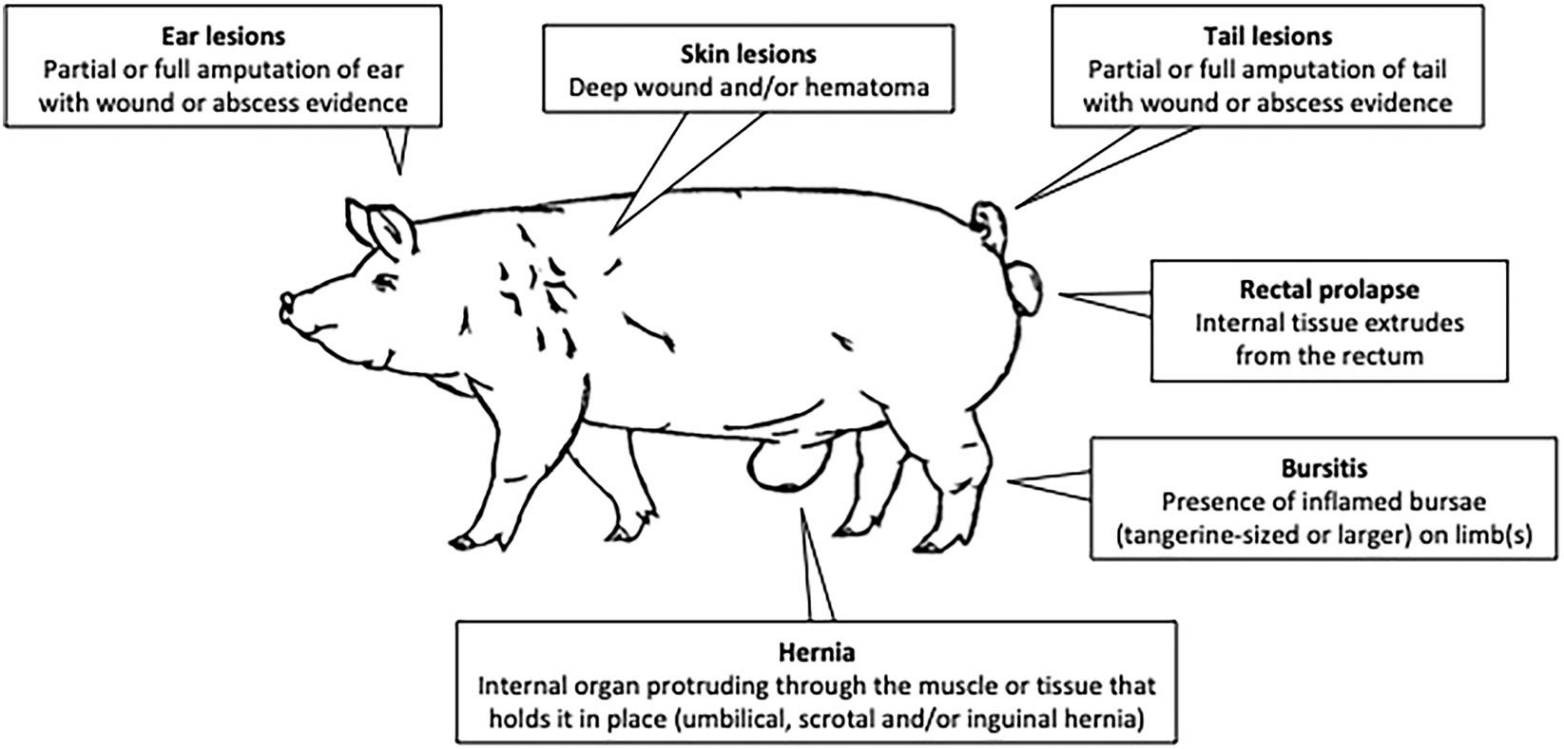
Welfare outcomes recorded at post‐mortem evaluation

At the first data collection point, groups of carcasses (range 12–35) were randomly selected in rounds of approximately 30 min, to ensure that the observer had enough time to move to the next point to continue with data collection. Each study carcass received an individual tattoo (slap number) on the shoulder for further carcass identification; the sex (female or castrated male) and the presence or absence of animal‐based welfare outcomes (ear, tail, and skin lesions, hernias, bursitis, and rectal prolapses) were recorded according to Teixeira et al. (2020) (Figure 1). Only severe cases of ear, tail, and skin lesions (i.e., evidence of puncture wound) were considered. It was not possible to record herd identification codes.

At the second data collection point, the reason and anatomical locations of carcass condemnations and carcass trimmings and associated disease lesions were recorded as present or absent on the basis of the decision of the VI on the slaughter line as per Teixeira et al. (2016) (Table 1). As the VI changed according to shifts, their identity (later codified from 1 to 5) was also recorded. VI 1 inspected 169 carcasses, VI 2 inspected 234 carcasses, VI 3 inspected 297 carcasses, VI 4 inspected 401 carcasses, and VI 5 inspected 399 carcasses. At the third data collection point, one observer recorded the line “kill number” of the study carcass in order to retrieve cold carcass weights from abattoir records.

**TABLE 1:** Definition of carcass condemnations and disease lesions associated with viscera condemnations detected at meat inspection

	Appearance/definition
Carcass condemnation detected at meat inspection	
Entire carcass	Removal of the entire carcass due to systemic disease
Partial carcass	Removal of part of the carcass due to disease or injury affecting hind limbs, forelimbs, or head
Trimmings	Removal of superficial or small part of the carcass (e.g., ear, tail, skin, hand, and feet) due to superficial disease/injury lesion/external abscess
Main disease lesions associated with carcass and viscera condemnations detected at meat inspection ^a^	
Abscess	Single or multiple focal, spherical, encapsulated purulent lesions
Viscera adherence	Adherence of the viscera to the abdominal or chest cavity
Tail lesion	Partial or full amputation of tail with wound or abscess evident
Fracture	Broken bone
Lesion	Wound or abscess evident
Nephritis	Inflamed nephrons (the functional units of the kidney)
Cysts	Presence of numerous, mostly small cysts located in the renal parenchyma

^a^ Only those disease lesions with more than five cases were considered. Pericarditis (*n* = 4), dermatitis (*n* = 5), edema (*n* = 4), bruise (*n* = 5), nephrosis (*n* = 1), pneumonia (*n* = 1), septicemia (*n* = 3), and tuberculosis (*n* = 2) were group as “other reasons.”

*Source*: Adapted from Teixeira et al. (2020) and Teixeira et al. (2016).

### 2.2 Phase 2: Animal‐based welfare outcomes and carcass temperature and pH

The second phase of the study was carried out during 12 slaughter days. Data were collected at two points on the slaughter line: (I) before dehairing and evisceration and (II) at the cold chamber.

At the first data collection point, groups of carcasses (range 9–68) were randomly selected. Each study carcass received an individual tattoo (slap number) on the shoulder for further carcass identification; the sex and the presence or absence of animal‐based welfare outcomes were also recorded. It was not possible to record herd identification codes. At the second data collection point, temperature and pH post‐mortem of the *m. longissimus* were determined using a portable temperature and pH meter (Mettler Toledo SevenGo™ Basic pH meter SG2), which was inserted into a small incision in the right loin (L2–L3 vertebrae). Measures were taken approximately 3 h after slaughter.

### 2.3 Technical notes

The first data collection point before dehairing and evisceration, rather than in lairage, was selected due human safety reasons (i.e., in lairage proved dangerous for the observers owing to fighting between the pigs and slippery floors) but also because it presents an ideal opportunity to measure the selected animal‐based welfare outcomes. Based on work by Carroll et al. (2016), we assumed that the animal‐based welfare outcomes would not be obscured by the stunning, exsanguination, and the scalding and dehairing processes.

It is important to note that in both phases, the observer positioned before dehairing and evisceration (point I) started the data collection when a member of the abattoir staff advised that animals from hospital pens were sending to slaughter. We adopted this strategy to increase the probability of collecting data from more animals with the selected welfare outcomes (Diana et al., 2019). Hence, the data do not represent the true prevalence of welfare lesions in slaughter pigs at this abattoir.

### 2.4 Statistical analysis

Descriptive analysis was calculated using Microsoft® Excel 2011 for Mac, and all other statistical analyses were conducted using SAS 9.3. Statistical associations were considered when *P* < 0.05 and tendencies when 0.05 < *P* < 0.1.

Generalized mixed models (Proc Glimmix) were used to analyze the association between sex and the presence of animal‐based welfare outcomes. Different models were built for each welfare outcome, with sex included as fixed effect, welfare outcomes as the dependent variables, and carcass as the experimental unit. A glogit link function was used with multinomial distribution specified. Results are presented as odds ratio (OR) and 95% confidence intervals (95% CI).

The relationship between animal‐based welfare outcomes and the number of carcass entire and partial condemned and/or trimmed and viscera condemnations was also analyzed using generalized mixed models (Proc Glimmix). Carcass condemnations (entire and partial), carcass trimmings, and viscera condemnations were considered as the dependent variable. First, univariable models were built to separately assess the influence the dependent variables. Predictor variables with *P* < 0.20 were used to build multivariate models. Backward selection was used to eliminate predictor variables until only those with *P* < 0.10 remained in the final model. VI and sex were forced into the models to assess their influence on the outcome variables. Due to the low prevalence of carcasses presenting ear lesions (*n* = 8) and rectal prolapses (*n* = 3), they were not considered for this statistical analysis. Results are presented as OR and 95% CI.

Weight, pH, and temperature of carcass were tested for normality before analysis using the Shapiro–Wilk test and examination of the normal plot. For the first phase of the study, the relationship between the sex, welfare outcomes, and carcass weight was assessed using generalized linear models (Proc Mixed). Sex and welfare outcomes were included as fixed effect and animal as the experimental unit. For this analysis, 116 carcasses were excluded as they presented more than one welfare outcome. Also, 255 carcasses were excluded as their weight was reduced because of being entirely or partially condemned and/or trimmed. Finally, 128 carcasses did not have the weight recorded and were also excluded. Due to the low prevalence of carcasses presenting ear lesions (*n* = 4) and rectal prolapse (*n* = 1), they were not considered for this statistical analysis.

For the second phase of the study, the relationship between the sex and welfare outcomes and the temperature and pH of the carcass was assessed using generalized linear models (Proc Mixed). Sex and welfare outcomes were included as fixed effects and animal as the experimental unit. For this analysis, 91 carcasses were excluded as they presented more than one welfare outcome. For the carcass temperature analysis, 24 carcasses were excluded due to missing data. Due to the low prevalence of carcasses presenting ear lesions (*n* = 0), hernia (*n* = 3), and rectal prolapse (*n* = 0), only tail and skin lesions and bursitis were considered for this statistical analysis. Results are reported as least square mean (LSM) ± standard error of the mean(SEM).

## 3 RESULTS

### 3.1 Descriptive results

In the first phase of the study, the final study population included carcasses from 781 male and 719 female pigs. General description of the study population, including the percentage of carcasses affected by each welfare outcome, location of condemnations and trimmings, and viscera condemnations, is in Table 2. Bursitis and skin lesions were the main welfare outcomes recorded, followed by tail lesions. Of the final population, 42 carcasses were fully or partially condemned. Nineteen carcasses were partially condemned, of which the head and the hindquarters were the most commonly affected anatomical regions. Carcass trimming occurred more frequently than condemnation; cumulatively 17% of the study population was either condemned or trimmed. The lungs and kidneys were most frequently condemned (16.7% in total).

**TABLE 2:** General description of the study population for the first phase of the study, including the percentage of carcass affected by each welfare outcomes, location of condemnations and trimmings, and reasons for viscera condemnations

		Total	% study population
Carcasses	Total	1500	100.0
	Female	719	47.9
	Male	718	52.1
Welfare outcomes	Total^a^	1259	83.9
	No lesions	241	16.1
	Ear lesions	8	0.5
	Tail lesions	245	16.3
	Skin lesions	376	25.1
	Hernia	54	3.6
	Bursitis	691	46.1
	Prolapse	3	0.2
Condemnations	Total	42	2.8
	Entire carcass	23	1.5
	Partial carcass	19	1.3
Partial condemnations^b^	Hindquarters	9	0.6
	Forequarters	1	0.0
	Head	9	0.6
Trimmings	Total^c^	213	14.2
	Tail	164	10.9
	Skin	28	1.9
	Feet	23	1.5
	Ham	4	0.3
	Ear	2	0.1
Viscera condemnations	Total^d^	258	17.2
	No viscera condemned	1242	82.8
	Lung	126	8.4
	Heart	78	5.2
	Liver	27	1.8
	Kidney	124	8.3

^a^ One hundred sixteen carcasses presented more than one welfare outcome.

^b^ Nineteen carcasses were both partial condemned and trimmed.

^c^ Sixteen carcasses had more than one part trimmed.

^d^ Eighty‐two carcasses had more than one viscus condemned.

For the second phase of the study, 316 carcasses were evaluated. General description of the study population, including the percentage of carcasses affected by each welfare outcomes, is in Table 3. As in the first phase, bursitis and skin lesions were more often recorded, and there were no cases of ear lesions or rectal prolapse.

**TABLE 3:** General description of the study population for the second phase of the study, including the percentage of carcass affected by each welfare outcome

		Total	% study population
Carcasses	Total	316	100.0
	Female	157	49.7
	Male	159	50.3
Welfare outcomes ^a^	Total	198	62.7
	No lesions	118	37.3
	Ear lesions	0	0.0
	Tail lesions	27	8.5
	Skin lesions	56	17.7
	Hernia	10	3.2
	Bursitis	151	47.8
	Prolapse	0	0.0

^a^ Forty‐five carcasses presented more than one welfare outcome.

### 3.2 Sex effect on welfare outcome results

In the first phase of the study, tail lesions affected males more frequently than female pigs (OR = 1.469; 95% CI 1.112–1.942; *P* < 0.01). However, male sex was not a risk factor for the other animal‐based welfare outcomes (*P* > 0.05). In the second phase of the study, sex was not a risk factor for any of the animal‐based welfare lesions outcomes evaluated (*P* > 0.05).

### 3.3 Carcass condemnation and trimming results

Tail lesions were a risk factor for entire carcass condemnations (OR: 11.379; 95% CI 3.472–37.286; *P* < 0.001) and trimmings (OR: 31.491; 95% CI 19.377–51.176; *P* < 0.001) but did not affect partial condemnations (OR: 1.406; 95% CI 0.410–4.818; *P* > 0.05). The other animal‐based welfare outcomes were not a risk factor for condemnations or trimmings, and neither was the VI or the sex of the pig (*P* > 0.05).

### 3.4 Viscera condemnation results

Hernia was a risk factor for kidney condemnations (OR: 2.470; 95% CI 1.148–5.312; *P* < 0.05). VI was also associated with heart and kidney condemnations (*P* < 0.05), but not lung and liver condemnations (*P* > 0.05).

### 3.5 Disease lesions results

A total of 502 cases of reasons for carcass and viscera condemnations were recorded, with 40 carcasses presenting more than one reason. From the cases identified, 1.4% of reasons was related to abscesses, 4.0% to multiple abscess, 17.3% to viscera adherence (excluding lung), 8.17% lung adherence, 37.4% to lesion, 6.0% to fracture, 24.1% to general lesions, 6.8% to nephritis, and 13.3% to cysts.

As only heart and kidney condemnations were affected by the presence of tail lesions and hernias, respectively, we explored the main reasons for condemning these two organs. From the 78 hearts condemned, 69 (88.5%) were due to viscera adherence; from the 124 kidneys condemned, eight (6.5%) were due to viscera adherence, 21 (16.9%) due to nephritis, and 64 (51.6%) due to cysts.

### 3.6 Cold carcass weight and carcass quality results

From the first phase of the study, there was a significant negative effect of tail lesions and hernias on carcass weight (*P* < 0.05; Table 4) such that there was an average reduction in weight of 3.6 (tail lesion) and 4.5 (hernia) kg relative to carcasses with no other welfare outcomes evaluated in this study. Also, carcasses from males (92.5 ± 0.52 kg) were heavier than those from female pigs (88.5 ± 0.52; *P* < 0.001).

**TABLE 4:** Least square mean (± SEM) weight (kg), pH, and temperature of carcass with none or one animal‐based welfare outcome and not condemned and/or trimmed

First phase of the study						
	Carcass weight					
Welfare outcomes	*n*	LSM (± SEM)	*P*‐value			
No lesions	189	90.8 (±0.84)				
Tail lesion	56	87.2 (±1.57)	0.0456			
Skin lesion	240	90.5 (±0.75)	0.8129			
Hernia	31	86.3 (±2.05)	0.0399			
Bursitis	514	90.9 (±0.53)	0.8754			

Second phase of the study						
	Carcass pH			Carcass temperature		
Welfare outcomes	*n*	LSM (± SEM)	*P*‐value	*n*	LSM (± SEM)	*P*‐value
No lesions	118	6.4 (±0.03)		106	13.5 (±0.18)	
Tail lesion	14	6.2 (±0.08)	0.0232	14	13.7 (±0.50)	0.6539
Skin lesion	25	6.4 (±0.06)	0.8176	25	14.4 (±0.42)	0.0511
Bursitis	110	6.3 (±0.03)	0.0036	99	14.3 (±0.21)	0.0044

*Note*: *P*‐values indicate significant differences between each welfare outcomes and no welfare outcome within columns.

From the second phase of the study, there was a significant negative effect of tail lesion and bursitis on carcass pH (*P* < 0.05); however, only the presence of bursitis affected carcass temperature (*P* < 0.05; Table 4). Finally, carcasses from males (6.4 ± 0.03) showed higher pH than those from female pigs (6.3 ± 0.03; *P* < 0.05). Similarly, temperature was higher in carcass from male (14.2 ± 0.19) than those from female pigs (13.6 ± 0.19; *P* < 0.05).

## 4 DISCUSSION

Data recording at abattoir ante‐mortem and post‐mortem meat inspections is a valuable way of assessing the health and welfare conditions that affect food‐producing animals. The present study found that, out of the animal‐based welfare outcomes evaluated in this study, tail lesions were a risk factor for entire carcass condemnations, reduced carcass weight, and pH of the carcass. The other welfare lesions evaluated were not associated with carcass condemnations, but the presence of a hernia affected carcass weight, and bursitis affected carcass pH and temperature. These results confirm previous findings that pigs affected by gross abnormalities have an increased risk of showing post‐mortem abnormalities (Harbers et al., 1992) and of having meat rejected at post‐mortem inspection (Jackowiak et al., 2006). Also, they confirm the hypothesis that ante‐mortem inspection can be used to predict post‐mortem outcomes in the same pigs, especially in cases of tail lesions and hernia, which might trigger attention of the VI in charge of the post‐mortem inspection.

This study did not aim to evaluate the prevalence of the selected animal‐based welfare outcomes. However, previous research carried out at the same abattoir (see Teixeira et al., 2020, for details) also found that bursitis and tail lesions were the most common welfare outcomes. It is important to note that any comparison with the prevalence of welfare lesions from the present study is not pertinent as batches with animals from hospital pens were purposely evaluated and included in this study to increase the probability of collecting data from more animals with the selected welfare outcomes and its association with post‐mortem outcomes. However, the association between the prevalence reported by Teixeira et al. (2020) and the carcass condemnations in the current study shows the magnitude of the tail biting behavior problem on farm, corroborating findings from previous studies in Finland and Ireland (Harley et al., 2014; Valros et al., 2004). However, similar to Harley et al. (2014), our findings do not show an association between tail lesions and lung condemnations or carcass abscessation. This was unexpected as the majority of papers shows such an association between tail lesions and these post‐mortem outcomes (Heinonen et al., 2010; Huey, 1996; Teixeira et al., 2016; Valros et al., 2004; van Staaveren et al., 2016) a potential mechanism for which was outlined by Boyle et al. (2022).

Carcasses from pigs affected by tail lesions showed a reduction in weight of 3.6 kg, corroborating previous studies (Harley et al., 2014; Valros et al., 2013; Vom Brocke et al., 2019). In fact, Harley et al. (2014) reported a reduction in carcass weight of 1.2, 3.27, and 12 kg in cases of mild, moderate, and severe tail lesions, respectively. Although we did not differentiate the severity of tail lesions, the finding associated with the reduction in carcass weight supports the association between tail lesion and the reduction in animal growth performance, which could be due to inflammatory processes (Boyle et al., 2022; Teixeira, Boyle, & Enríquez‐Hidalgo, 2020; Valros et al., 2004) or pain and stress in the victims of tail biting (Schrøder‐Petersen & Simonsen, 2001).

Carcasses from pigs affected by tail lesions also showed a lower muscle pH than carcasses with no other welfare outcomes, contradicting Valros et al.'s (2013) findings. The lower muscle pH of carcasses with tail lesions could be explained by the stress associated with tail biting (Taylor et al., 2010). It is possible that tail‐bitten pigs are more prone to psychological and/or physical stress just before slaughter (Valros et al., 2013) and, therefore, have higher blood lactate concentration at slaughter, which in the end is associated with lower initial muscle pH value (Dokmanovic et al., 2015). This might also explain the lower muscle pH value in animals affected by bursitis. In these cases, bursitis also affected muscle temperature, which again might be related to stress in animals affected by this condition (Schäfer et al., 2002).

Our study corroborated findings of previous studies showing that male sex increases the risk of tail lesions (Kritas & Morrison, 2007; Sinisalo et al., 2012; Valros et al., 2004), independent of whether they are entire (Harley et al., 2012, 2014; van Staaveren et al., 2016) or castrated (Hunter et al., 1999; Valros et al., 2004). Mixed sex groups were implicated in the reasons why males are at greater risk of being bitten (Boyle et al., 2022). One reason for the postulated higher propensity of females to bite than to be bitten could be that males in the pen out compete them for access to food and that they then employ tail biting to displace the males from the feeder/trough. In fact, the heavier carcasses from male compared with female pigs supports this theory. This finding is in line with Gispert et al. (2010) and is possibly explained by a greater average daily gain of castrated male pigs during the fattening period relative to female pigs (Fàbrega et al., 2010). However, Teixeira and Boyle (2014) found that entire males were heavier than female pigs prior to transport to the abattoir but there was no effect of sex on carcass weight, suggesting a higher weight of gut contents but similar kill out weight.

Carcasses from male pigs also presented higher carcass pH and temperature than carcasses from females which contrasts with Gispert et al. (2010), at least in regard to pH of the *Longissimus* muscle. Even though there was no association between sex and carcass condemnations, it is probable that the increased carcass pH in males is linked to their higher likelihood to have tail lesions. However, further studies with larger numbers of animals are needed to confirm this.

The presence of a hernia was associated with kidney condemnations, which differs from Ghidini et al. (2021) who found no association between kidney lesions and umbilical hernias. Also, carcasses from pigs affected by hernias were about 4.5 kg lighter relative to carcasses with no other welfare outcomes. Although growth weights from weaning to about 45 kg did not differ, weight gain prior to weaning is lower in pigs that develop hernias (Searcy‐Bernal et al., 1994), which could help to explain the lighter carcass weight relative to carcasses with no other welfare outcomes.

VI was also associated with heart and kidney condemnations. This finding confirms that the veterinary inspection can cause variation in the post‐mortem outcome. Such inconsistences have major implications for the use of post‐mortem inspection outcomes in disease surveillances. Common educational background, standardization of meat inspection criteria, and automated systems could help to mitigate these issues (Harley et al., 2012; Heinonen et al., 2021).

From the animal‐based welfare outcomes evaluated in this study, only tail lesions were associated with entire carcass condemnations and trimmings, a reduction in carcass weight, and a potential to impair carcass quality by reducing muscle pH, especially in carcasses from male pigs. Additionally, hernias were associated with viscera condemnation and a reduction in carcass weight. Therefore, our findings confirm that ante‐mortem inspection could be useful to predict post‐mortem outcomes in the same pigs, especially in cases of tail lesions and hernia, which might trigger attention of the VI in charge of the post‐mortem inspection.

These findings again illustrate the magnitude of the impact of tail biting on pig welfare and the health status of the herd on farm (Boyle et al., 2022). Raising animal health and welfare standards on farms, with concurrent improvements in animal performance (Nielsen, 2011), would promote a better image of the pig industry and inspire consumer confidence in the health and safety of pig meat.

## CONFLICT OF INTEREST

The authors declare that the research was conducted in the absence of any commercial or financial relationships that could be construed as a potential conflict of interest.
